# Intracellular assembly and budding of the Murine Leukemia Virus in infected cells

**DOI:** 10.1186/1742-4690-3-12

**Published:** 2006-02-10

**Authors:** Laurent Houzet, Bernard Gay, Zakia Morichaud, Laurence Briant, Marylène Mougel

**Affiliations:** 1 Laboratoire Infections Rétrovirales et Signalisation Cellulaire, CNRS UMR5121, UMI, IFR122, Institut de Biologie, Montpellier, France

## Abstract

**Background:**

Murine Leukemia Virus (MLV) assembly has been long thought to occur exclusively at the plasma membrane. Current models of retroviral particle assembly describe the recruitment of the host vacuolar protein sorting machinery to the cell surface to induce the budding of new particles. Previous fluorescence microscopy study reported the vesicular traffic of the MLV components (Gag, Env and RNA). Here, electron microscopy (EM) associated with immunolabeling approaches were used to go deeply into the assembly of the "prototypic" MLV in chronically infected NIH3T3 cells.

**Results:**

Beside the virus budding events seen at the cell surface of infected cells, we observed that intracellular budding events could also occur inside the intracellular vacuoles in which many VLPs accumulated. EM in situ hybridization and immunolabeling analyses confirmed that these latter were MLV particles. Similar intracellular particles were detected in cells expressing MLV Gag alone. Compartments containing the MLV particles were identified as late endosomes using Lamp1 endosomal/lysosomal marker and BSA-gold pulse-chase experiments. In addition, infectious activity was detected in lysates of infected cells.

**Conclusion:**

Altogether, our results showed that assembly of MLV could occur in part in intracellular compartments of infected murine cells and participate in the production of infectious viruses. These observations suggested that MLV budding could present similarities with the particular intracellular budding of HIV in infected macrophages.

## Background

Retroviruses consist of an enveloped capsid containing a dimer of genomic RNA. Genomic RNA contains genes encoding Gag, Gag/Pol and Env precursor proteins. The polyprotein Gag is sufficient for driving virus particle production by promoting assembly of immature capsid to the cellular membrane, budding, and release of the virus particles. The standard model for retrovirus production describes the budding of particles at the plasma membrane [1]. Addressing of Gag to the plasma membrane is promoted by the Matrix domain [2] and release of the newly formed particles from the cellular membrane is driven by the conserved "late domain" present in the Gag polyprotein of all retroviruses [3]. Integrity of the L-domain sequences is required for the late membrane fission event and the final pinching off of the budding virus [4-6]. In the last few years, late domain sequences were found to direct the interaction between the Gag proteins and some cellular factors involved in the protein sorting process and the vesicle formation during the multivesicular bodies (MVB) biogenesis [7,8]. MVB are late endosomal compartments accumulating internal vesicles produced from intracisternal invagination of the endosomal membrane. These internal vesicles are released either in lysosomes to allow associated protein and lipid degradation or in the extracellular space as exosomes for intercellular communication [9]. Internal vesicles production and virus budding are topologically similar processes consisting of budding away from the cytosol. Moreover, vacuolar protein sorting factors are involved in both events. These observations support the hypothesis that the virus hijacks the MVB production system to direct the budding and the release of virus particles [10].

Recently, it was shown that HIV and MLV Gag polyproteins can lead to the formation of virus-like particles (VLPs) in late endosomes [11]. Interestingly, intracellular-formed particles are the principal source of infectious HIV particles in infected macrophages [12]. These observations have led to the actual consideration of two pathways for HIV production: the standard budding at the plasma membrane and a new endosomal pathway [13]. In this latter, the fusion of the endosomes with the plasma membrane leads to virus particles release in the extracellular space [12].

Using fluorescence microscopy, several works reported the traffic of MLV Gag and Env proteins [11,14-16]] and viral genomic RNA [14] in endosomes of transfected or chronically infected cells. Here, we investigated virus assembly in NIH3T3 cells chronically infected with the replication-competent MLV using electron microscopy (EM). We showed that intracellular virus budding could arise and that numerous VLPs containing MLV genomic RNA accumulated in the Lamp-1 positive vacuoles. The absence of VLPs in lysosomal degradative compartments and the detection of intracellular infectious activity suggested that these intracellular virus particles could participate in the MLV infection.

## Results

### Intravacuolar virion-like particles in cells infected with the replication-competent MLV

**Figure 1:**
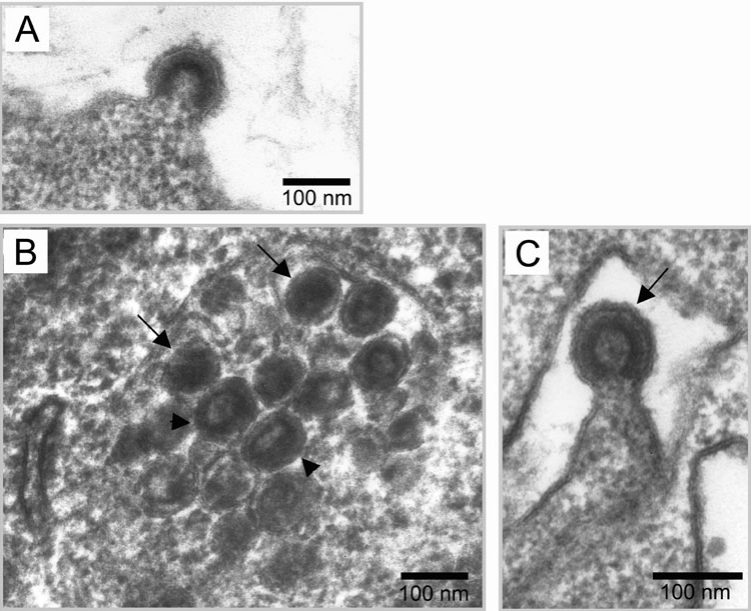
**Electron microscopy analysis of VLPs assembly in vacuoles of MLV-infected NIH3T3 cells. **EM analysis of epon embedded NIH3T3 cells chronically infected with MLV. A) Virus budding at the plasma membrane. B) Numerous mature (arrows) and immature (arrowheads) particles inside the intracellular endosomes. C) Budding particle into a vacuole.

MLV assembly was investigated by EM analysis in chronically infected NIH3T3 cells producing 10^5^-10^6 ^FFU per ml of cell culture supernatant. The use of chronically infected cells precludes reinfection with virion entry and ensures that only late phases of the viral cycle were observed. For cell morphology analysis, the cells were included in epon as previously described [17]. Rare budding viruses were detected at the plasma membrane (Fig. 1A), with only two budding events for hundred of analyzed cell-sections. In contrast, a large amount of particles with virus-like morphology were detected in intracellular vacuoles (Fig. 1B). The average size of these particles (90 nm diameter) corresponds to MLV particles. Moreover, the presence of dark electron dense ring or circle in these particles is typical of assembled MLV particles and corresponds respectively to immature and mature forms of capsids [18]. Noteworthy, several intravesicular buddings were also observed (Fig. 1C), with similar frequency as that observed for the external budding (2 events for hundreds of observed infected cells). These results indicated that intracellular budding of VLPs did occur in intracellular compartments of chronically infected cells.

### Identification of the intracellular VLPs by EM immunolabeling

To further characterize intracellular VLPs in the infected cells, we used EM approach coupled to immunolabeling with an anti-Gag antibody on lowicryl embedded sections. To estimate the frequency and intensity of the labeling on particles, we quantitated the number of labeled particles with the associated gold dots. Particles were identified by their size (between 90 and 100 nm diameter) and electron density criteria.

**Figure 2:**
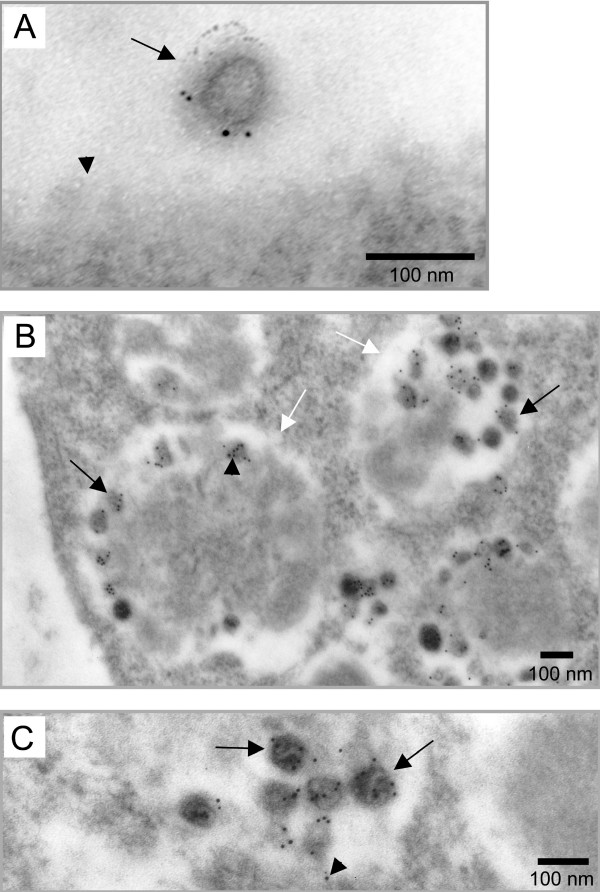
**Immunoelectron microscopy analysis of Gag distribution in MLV-infected cells and progeny viruses. **Gag was detected by immunogold labeling in lowicryl embedded sections of MLV-infected cells. A) Extracellular virus particle (black arrow) released from the plasma membrane (black arrowhead) labeled with 5 nm gold particles. B) VLPs (black arrows) present in intracellular vacuoles (white arrows) were labeled with similar intensity as extracellular viruses. C) Magnification of intravacuolar Gag-labeled VLPs (arrows). Weak labeling was also observed on the vacuolar delimiting membrane (arrowhead).

Due to the experimental procedure, very few extracellular virus particles were detected (7 for hundreds of infected cells). As expected, all were labeled for Gag antigens (Fig 2A), with an average intensity of 3,3 gold dots per virus (Table 1). Since virus particles were all immature and located in close vicinity of the plasma membrane, they probably have been just released from the plasma membrane.

**Table 1:** Quantification of anti-Capsid signal

	labeled particles (%)	Average labeling intensity (gold dots per particle)
extracellular	100	3,3 ± 1,5
intracellular	88	4,0 ± 2,5

The number of gold dots present on particles that displayed the typical morphology and size of virus particles was determined. Due to their very low abundance, only 7 external particles were analyzed, while a total of 52 intracellular particles were examined.

In the cells, very few labeled-Gag localized individually at the plasma membrane and most of the Gag proteins were detected in the intravacuolar VLPs (Fig. 2B, C). Despite the lower quality inherent in the immunolabeling procedure, all these Gag-labeled particles displayed the size and electron density characteristic of MLV particles and correlated to the VLPs observed before in epon embedded samples (Fig. 1B). Quantification of the labeling showed that 88% of the analyzed intravacuolar particles were labeled, with an intensity of 4 gold dots per particle, close to the labeling intensity observed for released viruses (Table 1). The unlabeled 12% could probably correspond to cellular vesicles which were identified by mistake as VLPs because of the non-optimal resolution in these assays. A weak Gag labeling was also observed on the vacuolar delimiting membrane (Fig. 2C), supporting our observation that the intravacuolar particles originated from budding of the delimiting membrane. These results indicated that intracellular VLPs observed in MLV-infected cells contained MLV Gag proteins and then corresponded to MLV virion-like particles.

### Encapsidation of the viral RNA genome in vesicular VLPs

**Figure 3:**
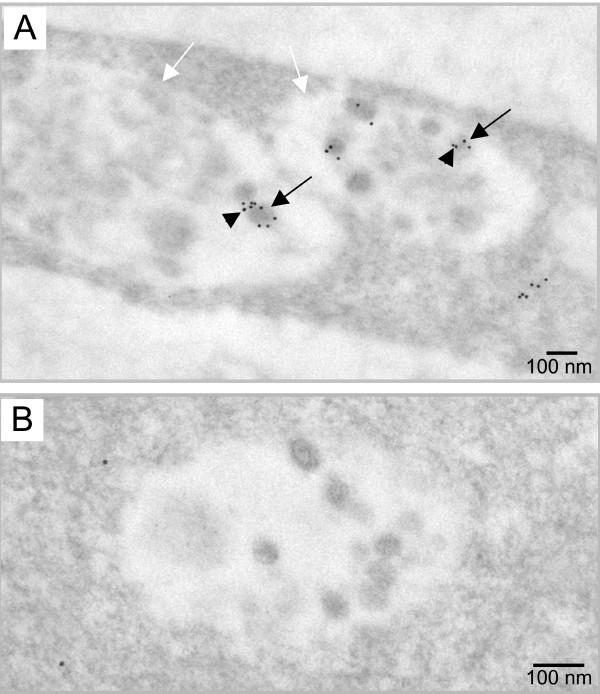
**Detection of the viral RNA genome in intracellular VLPs. **The MLV genomic RNA was specifically detected by EM in situ hybridization and was visualized by 10 nm gold particles. VLPs (black arrows) inside the intracellular vacuoles (white arrows) were labeled (arrowheads) with the specific antisense probe (A), while no signal was detected with the control sense riboprobe (B).

To go further in the analysis of the vesicular VLPs, presence of the genomic RNA was investigated by EM in situ hybridization with a specific DIG-labeled riboprobe. One difficulty of the labeling consists of the accessibility of the target sequence complementary to the probe, which must be exposed at the section surface to allow riboprobe hybridization. Among the 62 intracellular VLPs analyzed, 27 (44%) were labeled with the antisense riboprobe, showing that at least half of the internal VLPs contained genomic RNA (Fig. 3A). As expected, no particle labeling was observed with the sense riboprobe used as control (Fig. 3B). These results clearly showed that the viral RNA genome was packaged into the intracellular VLPs.

### Characterization of the VLP-containing vacuoles

**Figure 4:**
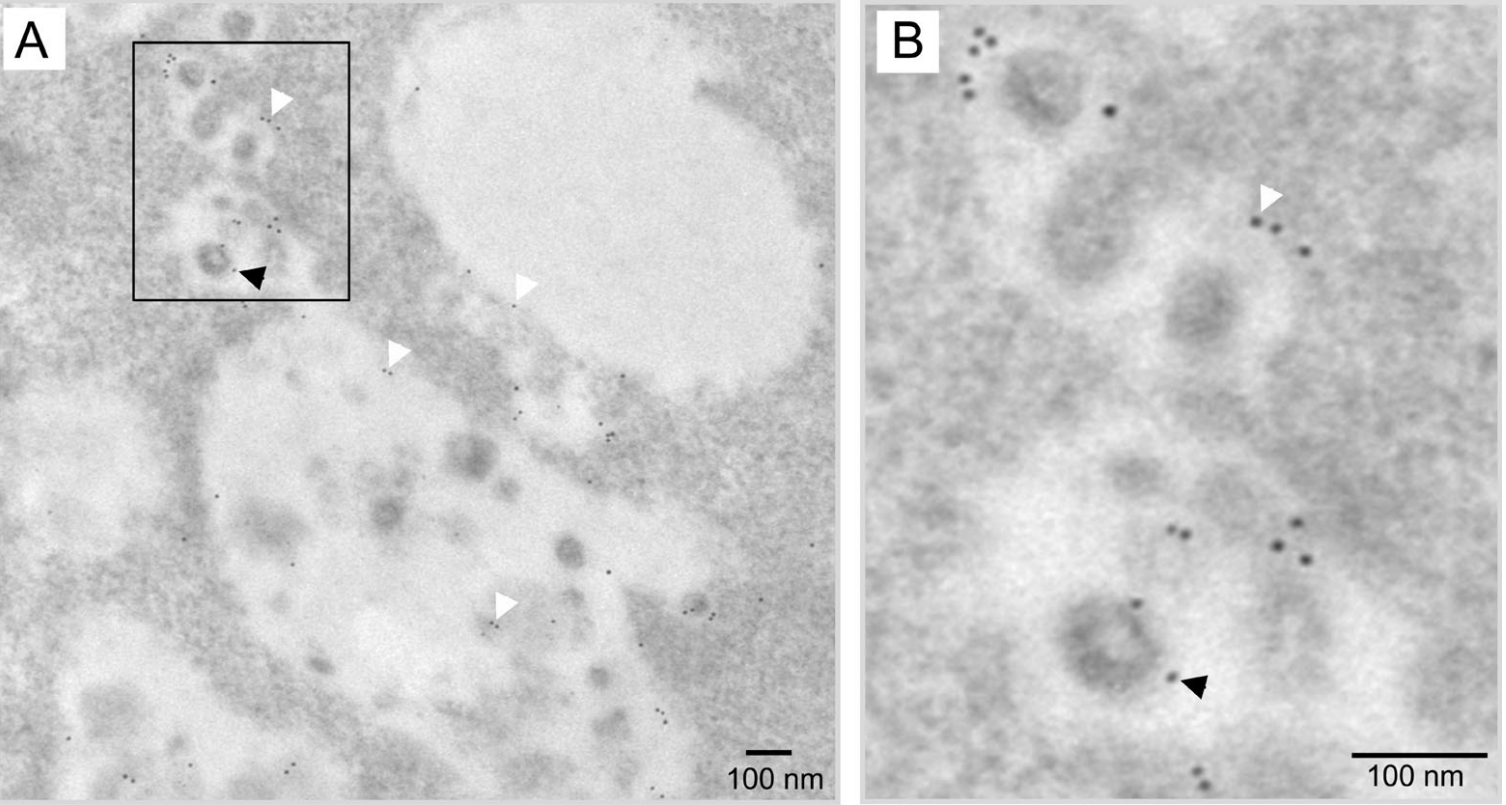
**VLP-containing vacuoles and their VLPs are positive for Lamp-1. **Lamp1 was detected in lowicryl embedded sections by immunogold labeling (5nm gold particles). A) Low labeling was observed on the periphery of MLV-VLPs containing vacuoles and on other intravacuolar components (white arrowheads). Gold particles could sometimes be found on individual intracellular MLV-VLP (black arrowhead). B) Magnification of the boxed area in A showed a Lamp1 positive MLV-VLP.

**Figure 5:**
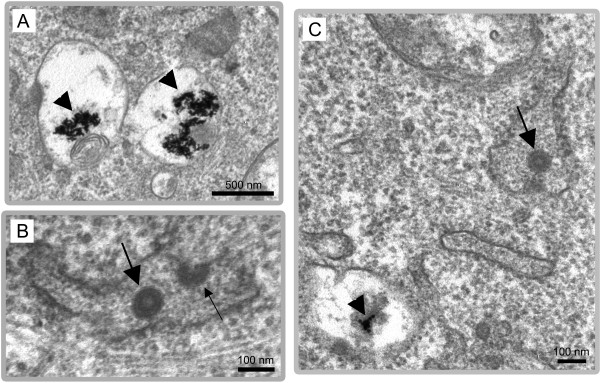
**Specific labeling of lysosomal compartments by pulses of BSA-gold. **Representative pictures of infected cells incubated with the BSA-gold. The BSA-gold accumulated in VLP-free lysosomes (arrowhead) (A). VLPs (large arrow) and budding event (little arrow) were shown in unlabeled vacuole (B). Absence of colocalization of VLP (arrow) and BSA-gold (arrowhead) (C).

In order to characterize the compartment which included the VLPs, immunolabeling experiments with an antibody directed against the late endosomal/lysosomal marker Lamp-1 were undertaken. A weak labeling was observed along the membrane of the vacuoles containing the VLPs, which is typical of a late endosome labeling (Fig. 4A) [12]. In addition, some VLPs present in these vacuoles also exhibited some low labeling (Fig. 4A and 4B). We noted that other non viral structures were labeled inside the vacuoles (Fig. 4A), which could correspond to the intracisternal vesicles of the MVB. To discriminate between late endosomes and lysosomes, lysosomal compartments were labeled by BSA-gold endocytosis. Infected cells were pulsed 4 hours with conjugates of BSA and 13-nm colloidal gold, chased for 20 hours to label lysosomes [19], and prepared for EM analysis. As expected, gold-labeled BSA exclusively accumulated in lysosomal compartments which appeared as white electron-light vacuoles (Figures 5-A and C). Clearly, the lysosome morphology differed from that of other vacuoles containing VLPs (Compare Fig 5A and 5B). More than hundred cells were analyzed and the colocalisation of the gold-labeled BSA and the VLPs was never observed in the same vacuole. Altogether, these results indicated that the intracellular compartments where the VLPs accumulated corresponded to the late endosomes related to MVB, and that no VLPs could be detected in lysosomes.

### Search for infectious activity in the lysate of MLV-infected cells

**Figure 6:**
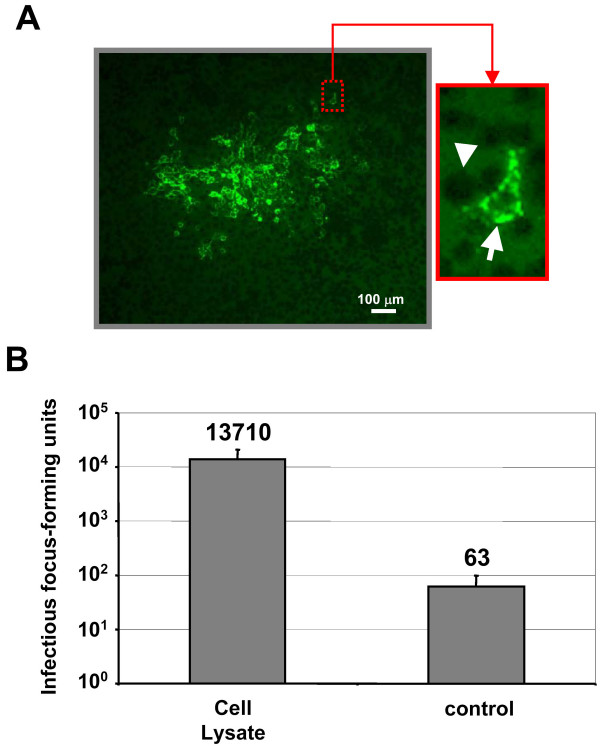
**Infectivity of intracellular particles released by freeze-thaw and sonication treatment. **FIA was used to quantitate infectious particles present in the cell lyzed by freeze-thaw and sonication (cell lysate) or in the last wash of cells left intact (control). A) One typical FFU labeled with anti-Env antibody and detected in FIA. Insert: magnification of the boxed area showing infected (arrow) and non infected (arrowhead) Dunni cells. B) Results of the FIA expressed as the total number of infectious FFU detected in the total lysate of 5 × 10^6 ^cells. Lysis and infectivity experiments were performed at least 3 times and each infection test was performed in triplicate. Bars, the standard error of the mean of each series.

Previous study reported that intracellular HIV particles in macrophages could harbor some infectious ability [20]. In order to test whether the intracellular MLV VLPs could also display some infectious ability, we undertook freeze and thaw experiments to release MLV related particles from the chronically infected cells. After drastic washes with cold PBS, 5 × 10^6 ^cells were lysed by several freeze-thaw cycles followed by a sonication step to release intracellular particles as described in Materials and Methods. Cellular debris were removed by centrifugation and filtration and the clarified cell lysate was used to infect target Dunni cells. Infectivity of intracellular particles was monitored by focal immunofluorescence assay (FIA) using an antibody specific to the MLV Env protein (Fig. 6A). As a control of wash efficiency, the same procedure was performed with the last wash supernatant of same cells left intact. The results are presented in Figure 6. Very little infectious activity was detected in the control assay that might result from residual contamination with external virus particles. However, a marked increase of the infectious activity was obtained when cells were submitted to the freeze-thaw and sonication lysis. The level of this intracellular activity is probably underestimated since virus particles contained in cell lysate could be damaged by the lysis procedure. Lysate obtained from mock-infected NIH3T3 did not show any infectious activity (data not shown). These results indicated the presence of intracellular infectious MLV particles in the chronically infected cells.

### Gag is sufficient to assemble vesicular VLPs

**Figure 7:**
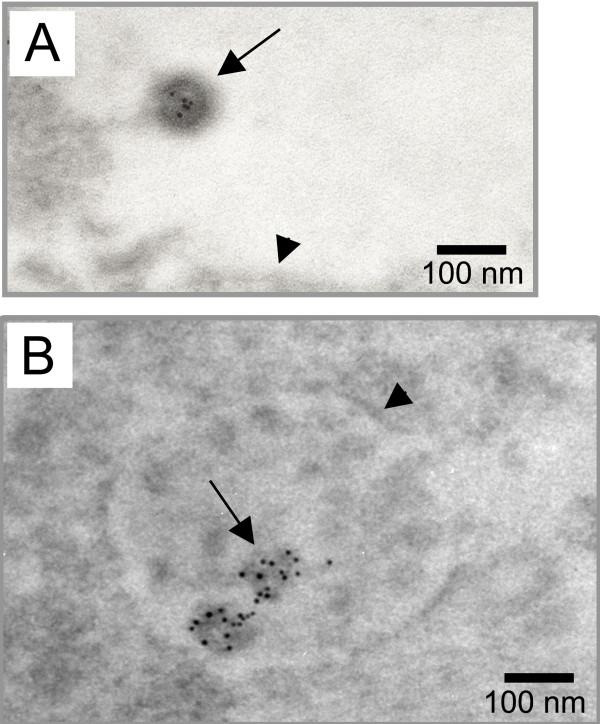
**Gag alone can promote intracellular VLPs formation. **Gag was detected by immunogold labeling in lowicryl embedded sections of Fly packaging cells which expressed the Gag and Pol proteins only. A) Extracellular VLP (arrow) released from the plasma membrane (arrowhead) labeled with 5 nm gold particles. B) VLPs (arrows) present in intracellular vacuole (arrowhead).

The Gag polyprotein is the basic component in the making of virion particles at the plasma membrane. Indeed, released VLPs can be obtained upon cellular expression of the sole Gag polyprotein [21] or can be assembled from purified Gag under certain conditions *in vitro *[22]. To investigate whether Gag may also promote the formation of the vesicular MLV particles, immunolabeling of Gag protein and EM analysis were conducted in derivative human HT1080 cells that expressed Gag/Gag-Pol alone (HT-Fly cells). As expected, a Gag-labeling of the external VLPs, recently detached from the plasma membrane was detected (Fig. 7A). Interestingly, we also noted the presence of intracellular VLPs displaying similar Gag-labeling (Fig. 7B). As observed with intravacuolar MLV particles in infected cells (Fig. 2B), these internal VLPs were concentrated (with some other unidentified vesicles) in intracellular compartments with a morphology that might correspond to this of MVB. Analysis of several cell sections revealed that Gag-VLP budded more frequently at the plasma membrane than at intracellular membrane. These observations differ somewhat with that observed in the context of chronic infection where frequencies of budding at the plasma membrane or in endosomes were similar. In conclusion, these results indicated that Gag alone was sufficient to generate not only the extracellular budding but also the formation of VLPs in intracellular compartment.

## Discussion

During the last years, most of the studies of endosomal traffic of retrovirus components were undertaken using fluorescence microscopy. Here we decided to take advantage of the high resolution of the EM to evaluate the assembly of the replication-competent MLV in chronically infected cells. Analysis of cellular content clearly showed that intracellular VLPs appeared very abundant in vacuolar compartments. This first observation substantiates previous study reporting VLPs in intracellular compartments in MLV-infected cells [23]. Using EM immunolabeling experiments, we identified them as MLV related VLPs. Several immunofluorescence studies reported that Env and Gag colocalized in intracellular compartments [11,14-16] and that a viral genomic RNA pool reaches the plasma membrane bound to Gag and Env tethered at the cytoplasmic face of the endosomal membrane [14]. The high resolution of the EM brings more precise results which clearly showed that, in the context of infection, a part of this genomic RNA pool was already encapsidated inside the intravesicular MLV particles and likely via intracellular budding events mediated by Gag. Furthermore, since the VLP-containing vacuoles were labeled by the late endosomal/lysosomal marker Lamp-1 and not by the BSA-gold, these latter could be identified as late endosomes. These results correlate with the visualization of MLV Gag in late endosomes by fluorescence microscopy [11,14].

We observed infectious activity in lysates of infected cells, indicating that infectious MLV particles were present inside the cells. It is tempting to speculate that these infectious virus particles corresponded to the MLV-VLPs we observed in late endosomes. Existence of intraendosomal virus budding and presence of many immature virus particles among these intracellular particles strongly suggested that these virus particles came from direct budding in the endosomal vacuoles. The frequency of intracellular budding events appeared low (2 for hundred of analyzed cell-sections) compared to the numerous particles accumulated in endosomes. However, in agreement with these results, Hansen et al [23] reported one intracellular VLP budding event for 22 analyzed cell-sections of MLV chronically infected cells. Similar observations were previously reported for the well documented intracellular HIV budding in macrophages where about 100 virions per vacuole were observed with only occasional budding events. This accumulation suggested that budding detection was probably dependent of the budding rates which drastically differs among the viruses [24,25] and which should be faster than the rate of the particles release.

The intraendosomal budding observed in the present study, in the context of the infection with the replication-competent MLV, might be a common alternative process shared by all retroviruses, since it was also documented in HIV infected macrophages [12].

Because late endosomes/MVB are directly linked to degradative pathway by fusion with lysosomal compartments, intraendosomal virus particles could be directly routed for degradation and not participate in virus production process. But the absence of detectable particles or viral components in lysosomes suggests that virus particles could escape the degradation pathway. Then, one can speculate that intracellular particles could be released in extracellular medium by fusion of the endosomal membrane with the plasma membrane and participate in MLV infection, as proposed for HIV in macrophages [12,20]. Thus, the virus particles production might occur from two different but non exclusive ways in MLV-infected NIH3T3 cells: the classical budding at the plasma membrane, and the budding into MVB. These budding events were both detected in cells expressing only Gag, suggesting the recruitment of a similar mechanism promoted by Gag.

It is not clear what determines the incidence of intracellular versus cell surface assembly. Indeed, numerous EM analyses performed with transfected MLV Gag or reconstituted viruses, usually in human (293T), monkey (Cos), or hamster (BHK21) cell lines, described exclusive budding at the plasma membrane [26-28]. At the opposite, only two studies (ours and [23]) showed the coexistence of intracellular and cell surface in chronically infected cells. One possible explanation for these different results is the experimental system : transient versus stable expression systems. The chronic phase of infection could favor the intracellular assembly as reported by Orenstein et al who compared HIV assembly during acute and chronic phases of infection [29]. Nevertheless, a recent work of Sherer et al showed that even in Gag-MLV transfected 293T and HeLa cells, MLV-VLPs can bud both at the plasma membrane and at the late endosomal membrane [11]. In addition, it cannot be excluded that the incidence of MLV intracellular versus cell surface assembly is also largely dependent of the cell lines as well documented in the case of HIV. In this latter, the plasma membrane budding was predominantly observed in T cells whereas virions accumulated in MVB in macrophages (see reviews [30-32]).

Recently, the observation of HIV intraendosomal budding and the discovery of the impact of endosomal proteins sorting pathway in retroviral budding have lead to the Trojan exosome hypothesis [33]. This original hypothesis proposes that retroviruses hijack the cellular exosomal production machinery leading to the production of exosome-like virus particles in the MVB and their release into the cell culture supernatant. Our report of similar alternative of intraendosomal budding in MLV-infected cells participates to a better understanding of the fundamental process involved in this late phase of retroviral infection. Moreover, MLV infection could constitute a new valuable model to evaluate *in vivo *the effect of new therapeutic agents directed against intraendosomal virus production.

## Materials and methods

### Cell culture and infection

NIH3T3, Dunni, and Fly (a kind gift from FL Cosset) cells were cultured in Dulbecco's modified Eagle's medium (DMEM) supplemented with glutamine (2 mM), penicillin, streptomycin and 10% heat-inactivated fetal calf serum at 37°C. Infections were performed with Friend-MLV viral stocks with average titer of 5 × 10^5 ^focus-forming units per ml (FFU/ml) as previously described [34]. MLV-infected NIH3T3 were maintained 1 month after infection and considered as chronically infected.

### EM and immuno-EM

For conventional EM, MLV-infected NIH3T3 cell samples were processed and embedded in epon (Embed-812, Electron Microscopy Sciences Inc.) according to a previously described method [17]. For immuno-EM, cells were fixed in 2,5% formaldehyde in 0.1M phosphate-buffered saline (PBS), pH7.4 for 90 minutes, washed in PBS + 0.05M ammonium chloride one hour, gathered in fibrin clot, and embedded in methacrylate resin (Lowicryl K4M, Chemische Werke Lowi). Ultrathin sections were cut with a Reichert OMU2 ultramicrotome and collected with gold grids 300 mesh. After blocking 20 minutes in Tris buffered saline (TBS) proteined (20 mM Tris-HCl pH 8,2, 20 mM sodium azide, 0,1% Tween 20, 1% goat serum, 1% bovine serum albumin), immunogold labeling was performed by incubating sections overnight at 4°C with primary antibody diluted in proteined TBS and one hour at room temperature with diluted gold labeling secondary antibody. Then, the grids were stained 20 minutes with 2% uranyl acetate in water, air dried, and examined on a Hitachi H1700 electron microscope. The following antibodies were used: rat monoclonal anti-Gag antibody (H187, a kind gift from B. Chesebro) or rat monoclonal anti-lamp1 antibody (clone 1D4B, a kind gift from M. Vidal) with goat anti-rat antibody coupled to 5 nm gold particles (British Biocell International, Cardiff, UK).

### EM in situ hybridization

The Digoxigenin labeled RNA probes were prepared from a linearized Bluescript plasmid containing a 652 bp MLV genomic fragment (position 1181 to 1833 bp). In vitro transcription was performed in the sense or anti-sense orientation using a DIG RNA labeling kit (Roche). Digoxigenin labeled RNA were quantified as instructed by the manufacturer. After 10 minutes incubation in the pre-hybridization buffer (4 × SSC + 50% formamide) at 37°C, ultra-thin sections were incubated overnight at 37°C in moist chamber in hybridization solution (1 μg/ml Dig-labeled RNA probe in 40% formamide deionised, 10% sulfate dextran, 1 × Denhart solution, 4× SSC, 250 μg/ml tRNA, 250 μg/ml salmon sperm DNA). The grids were washed 5 minutes in 2 × SSC and washed three times 5 minutes in 0,2 × SSC/60 % formamide at 37°C and twice 5 minutes in 2 × SSC at room temperature.

Immunogold detection of the Dig-labeled riboprobe was performed using mouse anti-Dig antibody (Roche) and goat anti-mouse antibody labeled with 10 nm colloidal gold particle (British Biocell International, Cardiff, UK). The procedure was the same as described above for immuno-EM, except that the incubation with primary antibody was 90 minutes at room temperature.

### Labeling of lysosomes by BSA-gold endocytosis

Colloidal gold (13 nm) was prepared by trisodium citrate reduction of gold chloride [35]. The colloid was adjusted to pH 6.0 with 0,2 M K_2_CO3 and conjugated to sufficient BSA to afford protection from NaCl-induced flocculation. BSA-gold was harvested using ultracentrifugation protocols which yielded monodisperse preparations free of aggregates and unbound protein. The preparations were dialyzed against PBS and adjusted to an *A*520 of 1.5 with DMEM.

For lysosomes labeling, infected cells grown to 70% confluence in 6 wells plate were starved 2 hours in DMEM. After cells incubation at 37°C in 3 ml of DMEM containing 150 μl of BSA-gold solution for 4 hours, the cells were washed 3 times with PBS and incubated in conjugate-free medium for 20 hours as previously described [19], prior to fixation and processing for EM.

### Detection of intracellular infectious activity

For each experiment, 5 × 10^6 ^chronically infected cells, producing viral supernatant with average titer of 5 × 10^5 ^FFU/ml, were washed 2 times with 10 ml of ice-cold PBS, scraped with a rubber policeman and transferred to centrifuge tubes. Cells were washed 3 more times with 20 ml cold PBS, resuspended in 100 μl of PBS and subjected to 4 freeze-thaw cycles followed by 2 times 30 sec sonication. Total cell disruption was microscopically validated using trypan blue staining. As a control for wash efficiency, the same procedure was performed with the last wash of same cells left intact. The samples (cell lysate or control) were then centrifuged at 2400 rpm for 10 min at 4°C and the supernatants of the centrifugation were added to 6 ml of culture medium and filtrated (0,45μm). For infections, serial dilutions of samples were used to infect target Dunni cells. Infectious particles were detected and quantitated by FIA, using monoclonal antibody (H48, a kind gift from B. Chesebro) specific to Friend-MLV Env protein [36].

## Abbreviations

EM, electron microscopy; HIV, human immunodeficiency virus; MLV, murine leukemia virus; MVB, multi vesicular bodies; VLPs, virus-like particles; FIA, focal immunofluorescence assay; FFU, focus-forming unit.

## Competing interests

The author(s) declare that they have no competing interests.
